# Argan Oil-Mediated Attenuation of Organelle Dysfunction, Oxidative Stress and Cell Death Induced by 7-Ketocholesterol in Murine Oligodendrocytes 158N

**DOI:** 10.3390/ijms18102220

**Published:** 2017-10-23

**Authors:** Asmaa Badreddine, Amira Zarrouk, El Mostafa Karym, Meryam Debbabi, Thomas Nury, Wiem Meddeb, Randa Sghaier, Maryem Bezine, Anne Vejux, Lucy Martine, Stéphane Grégoire, Lionel Bretillon, Emmanuelle Prost-Camus, Philippe Durand, Michel Prost, Thibault Moreau, Mustapha Cherkaoui-Malki, Boubker Nasser, Gérard Lizard

**Affiliations:** 1 Team Bio-PeroxIL, ‘Biochemistry of the Peroxisome, Inflammation and Lipid Metabolism’ (EA7270)/University Bourgogne Franche-Comté/Inserm, 21000 Dijon, France; asmaa.badreddine@gmail.com (A.B.); karimex100@hotmail.com (E.M.K.); debbabi.meryam55@gmail.com (M.D.); thomas.nury@u-bourgogne.fr (T.N.); wimed2@yahoo.fr (W.M.); sg.randa@yahoo.fr (R.S.); bezzinemaryem@yahoo.fr (M.B.); anne.vejux@u-bourgogne.fr (A.V.); malki@u-bourgogne.fr (M.C.-M.); 2 Laboratory of Neuroscience and Biochemistry, Faculty of Science and Technology, University Hassan 1er, Settat 26000, Morocco; boubker_nasser@hotmail.com; 3 Lab. NAFS, ‘Nutrition—Functional Food & Vascular Diseases’ LR12-ES-05, University of Monastir, Monastir 5000, Tunisia; zarroukamira@gmail.com; 4 Faculty of Medicine of Sousse, Sousse 4002, Tunisia; 5 ‘Institut Préparatoire aux Etudes Scientifiques et Techniques’ (IPEST), Laboratory ‘Matériaux, Molécules et Applications’ (LMMA), University of Carthage, La Marsa, Tunis 2078, Tunisia; 6 Pasteur Institut, Lab. ‘Venoms & Therapeutic Biomolecules’, University Tunis El Manar, Tunis 1068, Tunisia; 7 Eye & Nutrition Research Group, CSGA, UMR 1324 INRA, 6265 CNRS, University Bourgogne Franche-Comté, 21000 Dijon, France; lucy.martine@inra.fr (L.M.); stephane.gregoire@inra.fr (S.G.); lionel.bretillon@inra.fr (L.B.); 8 Laboratoires Spiral, 21560 Couternon, France; camusprost@hotmail.fr (E.P.-C.); laraspiral@wanadoo.fr (P.D.); michelprost.spiral@wanadoo.fr (M.P.); 9 Department of Neurology, University Hospital/University Bourgogne Franche-Comté, 21000 Dijon, France; thibault.moreau@chu-dijon.fr

**Keywords:** argan oil, extra virgin olive oil, α-tocopherol, 7-ketocholesterol, mitochondria, lysosome, peroxisome, oxiapoptophagy, 158N murine oligodendrocytes

## Abstract

Argan oil is widely used in Morocco in traditional medicine. Its ability to treat cardiovascular diseases is well-established. However, nothing is known about its effects on neurodegenerative diseases, which are often associated with increased oxidative stress leading to lipid peroxidation and the formation of 7-ketocholesterol (7KC) resulting from cholesterol auto-oxidation. As 7KC induces oxidative stress, inflammation and cell death, it is important to identify compounds able to impair its harmful effects. These compounds may be either natural or synthetic molecules or mixtures of molecules such as oils. In this context: (i) the lipid profiles of dietary argan oils from Berkane and Agadir (Morocco) in fatty acids, phytosterols, tocopherols and polyphenols were determined by different chromatographic techniques; and (ii) their anti-oxidant and cytoprotective effects in 158N murine oligodendrocytes cultured with 7KC (25–50 µM; 24 h) without and with argan oil (0.1% *v*/*v*) or α-tocopherol (400 µM, positive control) were evaluated with complementary techniques of cellular and molecular biology. Among the unsaturated fatty acids present in argan oils, oleate (C18:1 n-9) and linoleate (C18:1 n-6) were the most abundant; the highest quantities of saturated fatty acids were palmitate (C16:0) and stearate (C18:0). Several phytosterols were found, mainly schottenol and spinasterol (specific to argan oil), cycloartenol, β-amyrin and citrostadienol. α- and γ-tocopherols were also present. Tyrosol and protocatechic acid were the only polyphenols detected. Argan and extra virgin olive oils have many compounds in common, principally oleate and linoleate, and tocopherols. Kit Radicaux Libres (KRL) and ferric reducing antioxidant power (FRAP) tests showed that argan and extra virgin olive oils have anti-oxidant properties. Argan oils were able to attenuate the cytotoxic effects of 7KC on 158N cells: loss of cell adhesion, cell growth inhibition, increased plasma membrane permeability, mitochondrial, peroxisomal and lysosomal dysfunction, and the induction of oxiapoptophagy (OXIdation + APOPTOsis + autoPHAGY). Altogether, our data obtained in 158N oligodendrocytes provide evidence that argan oil is able to counteract the toxic effects of 7KC on nerve cells, thus suggesting that some of its compounds could prevent or mitigate neurodegenerative diseases to the extent that they are able to cross the blood-brain barrier.

## 1. Introduction

Lipids are a group of various organic substances. They are non-polar molecules that are soluble in organic solvents but not in water and are formed of carbon, hydrogen, oxygen and sometimes phosphorus, nitrogen and sulfur. In infants, the fats in breast milk and its substitutes are essential for brain development, and lipids are the source of infant dietary energy. These lipids are mainly fatty acids (FA): monounsaturated (MUFA) and polyunsaturated (PUFA) fatty acids, cholesterol and complex lipids [1,2,3,4]. In adults, lipid intake comes from different foods and in particular from oils, butter and margarines. About 50–60% of the dry weight of the brain consists of lipids, which play a leading role in the development of the brain and in brain functions [5]. Currently, there is evidence that abnormal lipid metabolism (fatty acids and cholesterol) plays a role in neurodegenerative diseases [6]. These abnormalities, observed in major neurodegenerative diseases (Alzheimer’s disease, multiple sclerosis, amyotrophic lateral sclerosis and peroxisomal leukodystrophies), may be partly due to mitochondrial and peroxisomal dysfunction, since these two organelles contribute to the metabolism of fatty acids and cholesterol and are essential for lipid homeostasis [7,8]. Several lines of evidence support the hypothesis that saturated and trans fatty acids increase the risk of dementia [9,10], and that high intake of polyunsaturated or monounsaturated fatty acids decreases this risk [11]. In addition, dietary fat composition is an important factor in blood–brain barrier function and also for the blood cholesterol profile [12]. Cholesterol and 24S-hydroxycholesterol, which is formed from cholesterol via the Cytochrome P450 46A1(CYP46-A1) enzyme [13,14], are involved in the neuropathology of Alzheimer’s disease, and the primary genetic risk factor for Alzheimer’s disease is apolipoprotein E-ε4 [6]. Dysfunctions in mitochondria and lipid metabolism also seem to play a crucial role in neurodegeneration with brain iron accumulation [15]. Moreover, the quality of dietary fatty acids also appears to be involved in the control of food intake and may thus be involved in obesity and anorexia [16]. Therefore, based on numerous studies, dietary lipids appear to be essential for the healthy development and functioning of the brain, and some of them could prevent neurodegeneration and delay brain aging [17,18,19]. In this context, it is important to take into account the fatty acid and nutrient profiles of edible oils, and to know more about their biological activities. Thus, it is possible that the consumption of certain oils recognized for their beneficial effects on health such as argan oil, used in Morocco, and olive oil, widely associated with the Mediterranean diet, could have beneficial effects on the brain [20].

Argan oil is obtained from the argan fruit of *Argania spinosa* L., an endemic tree located mainly in southwestern Morocco [21]. Up to now, argan oil has been used as a natural remedy in traditional medicine, mainly in Morocco, for several centuries [22]. Argan and olive oils are rich in tocopherols, phytosterols, and unsaturated fatty acid, which makes them very interesting oils with respect to their actions on the risk factors of numerous diseases, mainly cardiovascular diseases, associated with hyperlipidemia, hypercholesterolemia, and hypertension [23,24,25,26]. Argan oil is also traditionally used for the treatment of skin infections and in cosmetics [27,28].

There is also recent evidence in animal models that argan oil may exhibit neuroprotection. In the pilocarpine model used to induce epilepticus in wistar rats, argan oil administered by oral gavage increased catalase activity and attenuated oxidative stress in rat hippocampus [29]. Argan oil administered by oral gavage was shown to have cytoprotective effects on the brain of Sprague Dawley rats treated with acrylamide to induce oxidative stress-related neutotoxicity. These protective effects were reported on mitochondrial function, the anti-oxidant system and the activities of NADPH-generating enzymes [30]. Argan oil has also been reported to attenuate genetic damage and emperipolesis in rats treated with acrylamide [31]. In addition, in the model of neurodegeneration induced by aluminum chloride in male wistar rats (2.5 years old), argan oil given by oral gavage (6% *w*/*w* of argan oil in the food) for 42 days was also able to attenuate the decrease in catalase activity and to stimulate glutathione peroxidase activity in the hippocampus and cortex [20].

The biological activities of argan oil are mainly attributed to its content in major antioxidant molecules, tocopherols (α- and γ-tocopherol) and polyphenols [32,33]. In addition, recent evidence also suggests that Coenzyme Q10 (CoQ10) and melatonin, also identified in argan oil, have antioxidant properties [33]. As tocopherols, polyphenols, CoQ10 and melatonin are able to prevent oxidative stress and mitochondrial and/or peroxisomal dysfunctions, which are considered major events in several neurodegenerative diseases [34,35], these biological properties could at least in part explain some of the neuroprotective effects of argan oil.

Thus, as argan oil, which contains numerous nutrients able to cross the blood-brain barrier (fatty acids, phytosterols, polyphenols, tocopherols, etc.), can prevent neurotoxicity in several animal models and stimulate the activity of several anti-oxidant enzymes in the brain, it was important to determine its impact at the cellular levels on nerve cells. To this end, the cytoprotective effects of argan oil from Agadir and Berkane were evaluated in vitro in 158N cells treated with 7KC, which is formed by auto-oxidation of cholesterol, and found at high levels in the plasma, cerebrospinal fluid and/or brain of patients with Alzheimer’s disease [36], multiple sclerosis [37], Nieman-Pick disease [38] and X-linked adrenoleukodystrophy (X-ALD) [39]. Even though the in vitro model used in the present study (murine oligodendrocytes 158N cultured without or with 7KC associated or not with natural or synthetic molecules or mixtures of molecules) does not include selection of the bioactive molecules present in argan oil by the blood–brain barrier, it can be considered discriminatory to identify natural and synthetic molecules (or mixtures of molecules, such as oils) able to prevent the toxic effects of 7KC, which is associated with major age-related diseases (including Alzheimer’s disease) and with several severe neurodegenerative diseases, such as multiple sclerosis and X-ALD [39,40,41,42,43]. Thus, in the present study: (i) the fatty acid, phytosterol, polyphenol, and tocopherol profiles of argan oils from Agadir and Berkane were established comparatively to the profiles of extra virgin olive oil from Tunisia; (ii) the antioxidant properties of argan oils were evaluated with the KRL (Kit Radicaux Libres) test and with the ferric reducing antioxidant power (FRAP) assay; and (iii) the ability of argan oil to prevent major toxic effects of 7KC (loss of cell adhesion, cell growth inhibition, increased plasma membrane permeability, mitochondrial, peroxisomal and lysosomal dysfunctions, and induction of oxiapoptophagy) was determined.

## 2. Results

### 2.1. Fatty Acid, Phytosterol, Tocopherol and Polyphenol Profile of Argan Oils from Morocco and Extra Virgin Olive Oil from Tunisia

The characteristics of argan oil in terms of quality and composition depend on numerous parameters: genetic and environmental factors (climate and altitude) but also the oil extraction process, soil type, the temperature, rain, drought, fruit maturity and the harvest year [20,21,44]. It is therefore important to determine the composition of argan oils and to establish whether they contain particular compounds and whether some of their compounds can have cytoprotective effects on appropriate cell and animal models. In the present study, we focused on the fatty acids, phytosterols, polyphenols and tocopherols present in argan oil. Whatever the geographic origin of the argan oils (Agadir and Berkane) studied, among the unsaturated fatty acids, oleate (C18:1 n-9) and linoleate (C18:2 n-6) were the most abundant, and the highest quantities of saturated fatty acids present were palmitate (C16:0) and stearate (C18:0) (Table 1) [45]. Compared with the extra virgin olive oil considered (Madhia, Tunisia) [35], the argan oils were richer in linoleate (C18:2 n-6) but poorer in oleate (C18:1 n-9) and linolenate (C18:3 n-3), while both argan oils and extra virgin olive oil contained high levels of palmitate (C16:0) and stearate (C18:0) (Table 1). Numerous phytosterols were often present at similar levels in the argan and extra virgin olive oils in the following order: schottenol > cycloartenol > β-amyrin > citrostadienol > Δ7-avenasterol > spinasterol > 24-methylen cycloartenol > Δ7-stigmasterol > Δ7-campesterol > campesterol > lupeol (Table 2). Among these phytosterols, schottenol, which was the most abundant, was only detected in argan oils, as were Δ7-stigmasterol, Δ7-campesterol, spinasterol and lupeol (Table 2). These compounds are of interest for quality control, in particular for the identification of adulterated argan oils. Unlike other oils [46,47], argan and extra virgin olive oils do not contain cholesterol (Table 2), thus increasing the dietary interest of these oils as they will not generate cholesterol oxides oxidized at C7 (mainly 7-ketocholesterol). These oxides are derived from the auto-oxidation of cholesterol (storage, heating) and are known for their cytotoxic activities [48,49,50]. α- and γ-tocopherols were the most abundant tocopherols: the highest level of α-tocopherol was detected in argan oil from Agadir, the lowest level in argan oil from Berkane; the highest level of γ-tocopherol was detected in argan oil from Berkane, the lowest level in argan oil from Agadir; δ-tocopherol was always present at low concentrations (Table 3). Tyrosol and protocatechic acid (not detected in extra virgin oil) were the only polyphenols detected in argan oils; hydroxytyrosol was only found in extra virgin olive oil (Table 4).

Numerous compounds present in argan oils, including phytosterols, polyphenols and tocopherols are able to prevent nerve cell dysfunctions that lead to neurodegeneration both in vitro and in vivo [51,52,53]. These earlier observations led us to evaluate the ability of argan oils to prevent the toxic effects of 7KC, which is associated with numerous age-related diseases [14], and has been identified at elevated levels in the brains of patients with Alzheimer’s diseases [36]. 7KC could contribute to neurodegeneration via its ability to induce numerous side effects associated with neurodegenerescence, including the overproduction of reactive oxygen species, and the induction of mitochondrial, lysosomal and peroxisomal dysfunction, and cell death [35,54]. The ability of argan oil to impair 7KC-induced side effects was compared to that of α-tocopherol used as positive control.

### 2.2. Comparison of the Antioxidant Properties of Argan Oils from Morocco and Extra Virgin Olive Oil from Tunisia Using Two Complementary Techniques: The KRL and FRAP Tests

The antioxidant properties of α-tocopherol, argan oil from Agadir, argan oil from Berkane and extra virgin olive oil (Madhia, Tunisia) were determined using two complementary methods: the KRL (Kit Radicaux Libres) and the Ferric Reducing Antioxidant Power (FRAP) tests. These two tests use Trolox as the reference and provide similar information for the different compounds studied (Table 5). Data obtained with α-tocopherol were in the range of those previously reported and allowed us to validate our data [35]. According to the two tests, the antioxidant properties of argan and extra virgin olive oils were in the same range.

### 2.3. Evaluation of the Effects of Argan Oils on 7-Ketocholesterol-Induced Cell-Growth Inhibition Evaluated with the Crystal Violet and MTT Tests and by Phase-Contrast Microscopy

**Figure 1:**
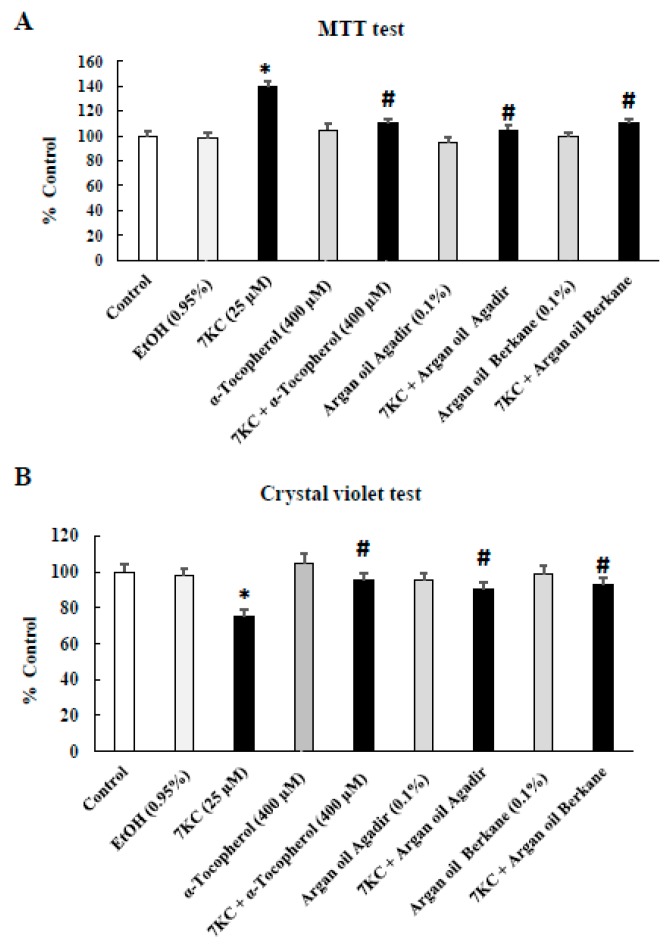
Evaluation of the effect of argan oil on 7-ketocholesterol (7KC)-induced cell growth inhibition in 158N murine oligodendrocytes with the crystal violet and MTT tests. After 24 h of culture, 158N murine oligodendrocytes were cultured for an additional 24 h without or with 7KC (25 µM) in the absence or presence of argan oils (Agadir or Berkane; Morocco; 0.1% *v*/*v*) or α-tocopherol (400 µM) used as the positive control. Argan oils and α-tocopherol were added to the culture medium 2 h before 7KC. The cytoprotective effect of argan oils on 7KC-induced inhibition of cell growth was evaluated with the crystal violet test (measurement of adherent cells) (**A**) or the MTT test (measurement of the activity of the succinate deshydrogenase, a mitochondrial enzyme belonging to the Krebs cycle) (**B**). The experiments were carried out three times in triplicate. Data are mean ± standard deviation (SD) of two independent experiments carried out in triplicate. The significance of the relationship between vehicle and cells treated with 7KC, argan oils or α-tocopherol was calculated by the Anova test (Sidak’s multiple comparisons); * *p* ≤ 0.05. The significance of the relationship between cells treated with 7KC alone, 7KC and argan oils cotreatment, or 7KC and α-tocopherol cotreatment was calculated by the Anova test (Sidak’s multiple comparisons); ^#^ *p* ≤ 0.05. No significant difference was found between control and vehicle-treated cells (EtOH 0.95% *v*/*v*). The EtOH value of 0.95% corresponds to the highest EtOH concentration obtained when the cells were simultaneously treated with 7KC used at 25 µM (EtOH, 0.05%) and with argan oil (EtOH, 0.9% *v*/*v*).

To determine the impact of argan oils (0.1% *v*/*v*, final concentration) on 7KC (25 µM, 24 h)-induced inhibition of cell growth, the crystal violet and the MTT ((3-(4,5-dimethylthiazol-2-yl)-2,5-diphenyltetrazolium bromide) tetrazolium) tests and phase-contrast microscopy were used. Under these conditions, the crystal violet test showed a significant decrease in adherent 158N cells following treatment with 7KC for 24 h (Figure 1A). In the presence of argan oils or α-tocopherol (400 µM) used as the positive control [35,55], this toxic effect of 7KC was significantly reduced (Figure 1A). The data obtained with the crystal violet test were in agreement with those obtained by phase-contrast microscopy, which revealed an inhibition of cell growth, associated with a smaller number of adherent cells and by an increased number of round cells, considered dead cells, floating in the culture medium (Figure S1) [56]. However, with the MTT test, which is also used to measure cell growth via the enzymatic activity of succinate deshydrogenase (a mitochondrial enzyme of the Krebs cycle), higher MTT values were observed following treatment with 7KC, suggesting a hyperpolarization process preceding major mitochondrial damage leading to the loss of transmembrane mitochondrial potential (ΔΨm) and cell adhesion [57,58] (Figure 1A). This hyperpolarization was corrected by argan oils and α-tocopherol (Figure 1A). Notably, no significant differences were observed between control (untreated cells), vehicle-treated cells (Ethanol (EtOH): 0.9%), argan oils and α-tocopherol on cell growth evaluated with the crystal violet test (Figure 1B). These data were in agreement with the observations made by phase-contrast microscopy (Figure S1). Similarly, no effects of EtOH, argan oils and α-tocopherol were found on succinate deshydrogenase activity determined with the MTT test (Figure 1A).

### 2.4. Evaluation of the Effects of Argan Oils on 7-Ketocholesterol-Induced Cell Overproduction of Reactive Oxygen Species: Measurement by Flow Cytometry after Staining with Dihydroethidine

**Figure 2:**
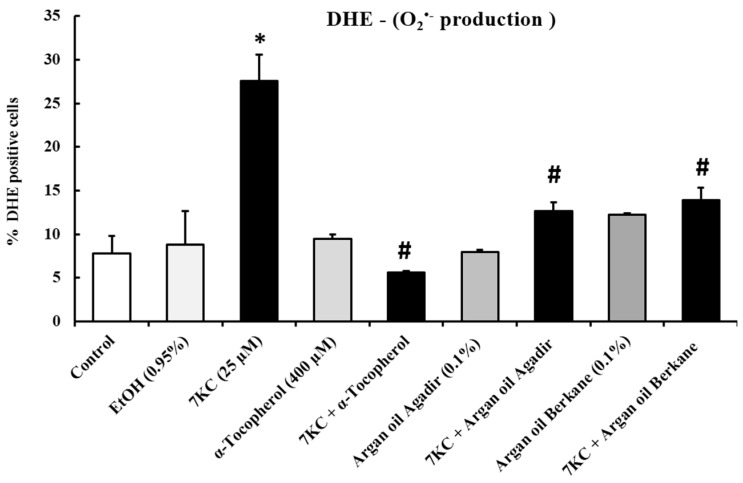
Evaluation of the effect of argan oil on 7-ketocholesterol-induced overproduction of reactive oxygen species in 158N murine oligodendrocytes by flow cytometry after staining with dihydroethidine. After 24 h of culture, 158N murine oligodendrocytes were cultured for an additional 24 h without or with 7KC (25 µM) in the absence or presence of argan oils (Agadir or Berkane; Morocco; 0.1% *v*/*v*) or α-tocopherol (400 µM) used as the positive control. Argan oils and α-tocopherol were added to the culture medium 2 h before 7KC. The cytoprotective effect of argan oils on 7KC-induced overproduction of reactive oxygen species (ROS), mainly superoxide anions, was evaluated by flow cytometry after staining with dihydroethidine (DHE). ROS overproduction was determined by the percentage of DHE-positive cells. The experiments were carried out twice in triplicate. Data are mean ± SD of two independent experiments carried out in triplicate. The significance of the relationship between vehicle and cells treated with 7KC, argan oils or α-tocopherol was calculated by the Anova test (Sidak’s multiple comparisons); * *p* ≤ 0.05. The significance of the relationship between cells treated with 7KC alone, 7KC and argan oils cotreatment, or 7KC and α-tocopherol cotreatment was calculated by the Anova test (Sidak’s multiple comparisons); ^#^ *p* ≤ 0.05. No significant difference between control and vehicle-treated cells (EtOH 0.95% *v*/*v*).

As 7KC is known to induce oxiapoptophagy (OXIdation + APOPTOsis + autoPHAGY) in 158N cells [59,60], the ability of argan oils to prevent oxidative stress in these cells was studied. The effects of argan oils (0.1% *v*/*v*, final concentration) and α-tocopherol (400 µM) on 7KC (25 µM, 24 h)-induced over-production of reactive oxygen species (ROS), mainly superoxide anions, were evaluated by flow cytometry after staining with dihydroethidine (DHE). In 158N cells, 7KC promoted ROS overproduction: in comparison with control and vehicle-treated cells, the percentage of DHE-positive cells following treatment with 7KC was markedly increased. Treatment with argan oils or α-tocopherol alone had no effect on ROS overproduction. When the argan oils and α-tocopherol were added with 7KC, the overproduction of ROS was strongly attenuated in comparison with 7KC treatment alone (Figure 2). No significant differences were observed between control or vehicle-treated cells (Ethanol (EtOH): 0.9%) and argan oil- or α-tocopherol-treated cells for ROS overproduction (Figure 2).

### 2.5. Evaluation of the Effects of Argan Oils on 7-Ketocholesterol-Induced Increased Plasma Membrane Permeability: Measurement by Flow Cytometry after Staining with Propidium Iodide

**Figure 3:**
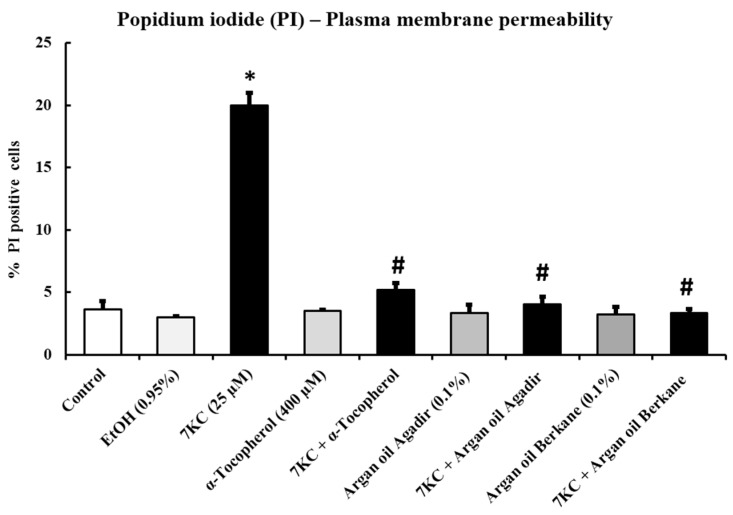
Evaluation of the effect of argan oil on 7-ketocholesterol-induced plasma membrane permeability in 158N murine oligodendrocytes by flow cytometry after staining with propidium iodide. After 24 h of culture, 158N murine oligodendrocytes were cultured for an additional 24 h without or with 7KC (25 µM) in the absence or presence of argan oils (Agadir or Berkane; Morocco; 0.1% *v*/*v*) or of α-tocopherol (400 µM) used as the positive control. Argan oils and α-tocopherol were added to the culture medium 2 h before 7KC. The cytoprotective effect of argan oils on 7KC-induced plasma membrane permeability was evaluated by flow cytometry after staining with propidium iodide (PI). Plasma membrane permeability was determined by the percentage of PI positive cells. The experiments were carried out twice in triplicate. Data are mean ± SD of two independent experiments carried out in triplicate. The significance of the relationship between vehicle and cells treated with 7KC, argan oils or α-tocopherol was calculated by the Anova test (Sidak’s multiple comparisons); * *p* ≤ 0.05.The significance of the relationship between cells treated with 7KC alone, 7KC and argan oils cotreatment, or 7KC and α-tocopherol cotreatment was calculated by the Anova test (Sidak’s multiple comparisons); ^#^ *p* ≤ 0.05. No significant difference was observed between control and vehicle-treated cells (EtOH 0.95% *v*/*v*).

The effects of argan oils (0.1% *v*/*v*, final concentration) and α-tocopherol (400 µM) on 7KC-induced (25 µM, 24 h) plasma membrane alterations were evaluated by flow cytometry after staining with propidium iodide (PI). In 158N cells, 7KC induced an increase in plasma membrane permeability (evaluated by the percentage of PI-positive cells), which could be promoted by ROS overproduction: compared with control and vehicle-treated cells, the percentage of PI-positive cells under treatment with 7KC was markedly and significantly increased. When the argan oils and α-tocopherol were added with the 7KC the increase in plasma membrane permeability was strongly attenuated, in comparison with 7KC treatment alone (Figure 3). Treatment with argan oils or α-tocopherol had no effect on plasma membrane permeability. Of note, no significant differences were observed between control or vehicle-treated cells (Ethanol (EtOH): 0.9%) and argan oil- or α-tocopherol-treated cells for ROS overproduction (Figure 3).

### 2.6. Evaluation of the Effects of Argan Oils on 7-Ketocholesterol-Induced Acidic Vesicle Formation: Measurement by Flow Cytometry after Staining with Acridine Orange

**Figure 4:**
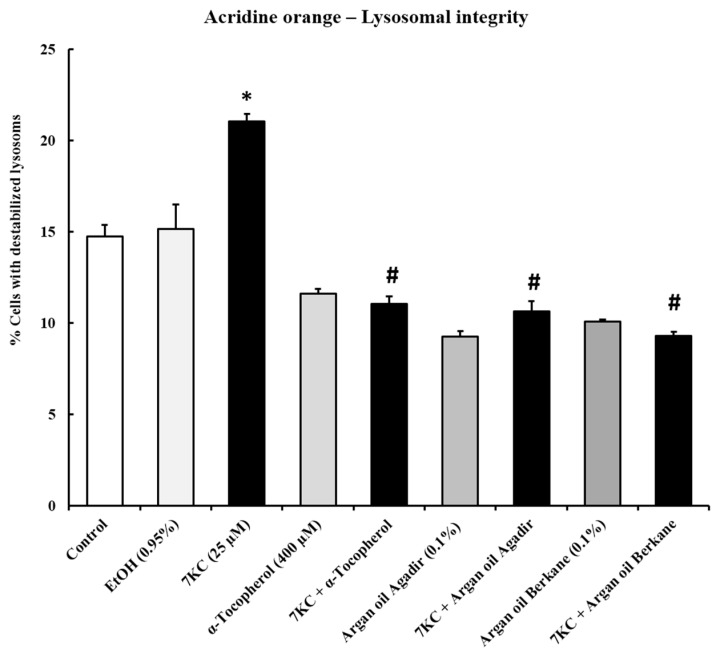
Evaluation of the effect of argan oil on 7-ketocholesterol-induced acidic vesicle formation in 158N murine oligodendrocytes by flow cytometry after staining with acridine orange. After 24 h of culture, 158N murine oligodendrocytes were cultured for an additional 24 h without or with 7KC (25 µM) in the absence or presence of argan oils (Agadir or Berkane; Morocco; 0.1% *v*/*v*) or of α-tocopherol (400 µM) used as the positive control. Argan oils and α-tocopherol were added to the culture medium 2 h before 7KC. The cytoprotective effect of argan oils on 7KC-induced acidic vesicle formation was evaluated by flow cytometry after staining with acridine orange (AO). Acidic vesicle formation was determined by the percentage of AO-positive cells. The experiments were carried out twice in triplicate. Data are mean ± SD of two independent experiments carried out in triplicate. The significance of the relationship between vehicle and cells treated with 7KC, argan oils or α-tocopherol was calculated by the Anova test (Sidak’s multiple comparisons); * *p* ≤ 0.05. The significance of the relationship between cells treated with 7KC alone, 7KC and argan oils cotreatment, or 7KC and α-tocopherol cotreatment was calculated by the Anova test (Sidak’s multiple comparisons); ^#^ *p* ≤ 0.05. No significant difference was detected between control and vehicle-treated cells (EtOH 0.95% *v*/*v*).

7KC-induced oxiapoptophagy is associated with an increase in acidic vesicle formation, which could correspond to autophagic vesicle formation [60]. This increase in numbers of acidic vesicles could also, at least in part, be an adaptive response to the alteration of lysosomes resulting from the accumulation of 7KC in these organelles, subsequently leading to lysosomal membrane damage [43]. The effects of argan oils (0.1% *v*/*v*, final concentration) and α-tocopherol (400 µM) on 7KC (25 µM, 24 h)-induced acidic vesicle formation was evaluated by flow cytometry after staining with acridine orange (AO). In 158N cells, 7KC induced an increase in acidic vesicle formation (evaluated by the percentage of AO-positive cells): in comparison with control and vehicle-treated cells, the percentage of AO-positive cells following treatment with 7KC was significantly increased (Figure 4). When the argan oils and α-tocopherol were added with 7KC, the increase in 7KC-induced acidic vesicle formation was strongly attenuated, compared with 7KC treatment alone (Figure 4). No significant differences were observed between control and vehicle-treated cells (Ethanol (EtOH): 0.9%); however, treatment with argan oils and α-tocopherol slightly decreased the formation of acidic vesicles (Figure 4).

### 2.7. Evaluation of the Effects of Argan Oils on 7KC-Induced Peroxisomal Dysfunction Evaluated by Abcd1, Abcd3, Acox1, and Mfp2 mRNA Levels

**Figure 5:**
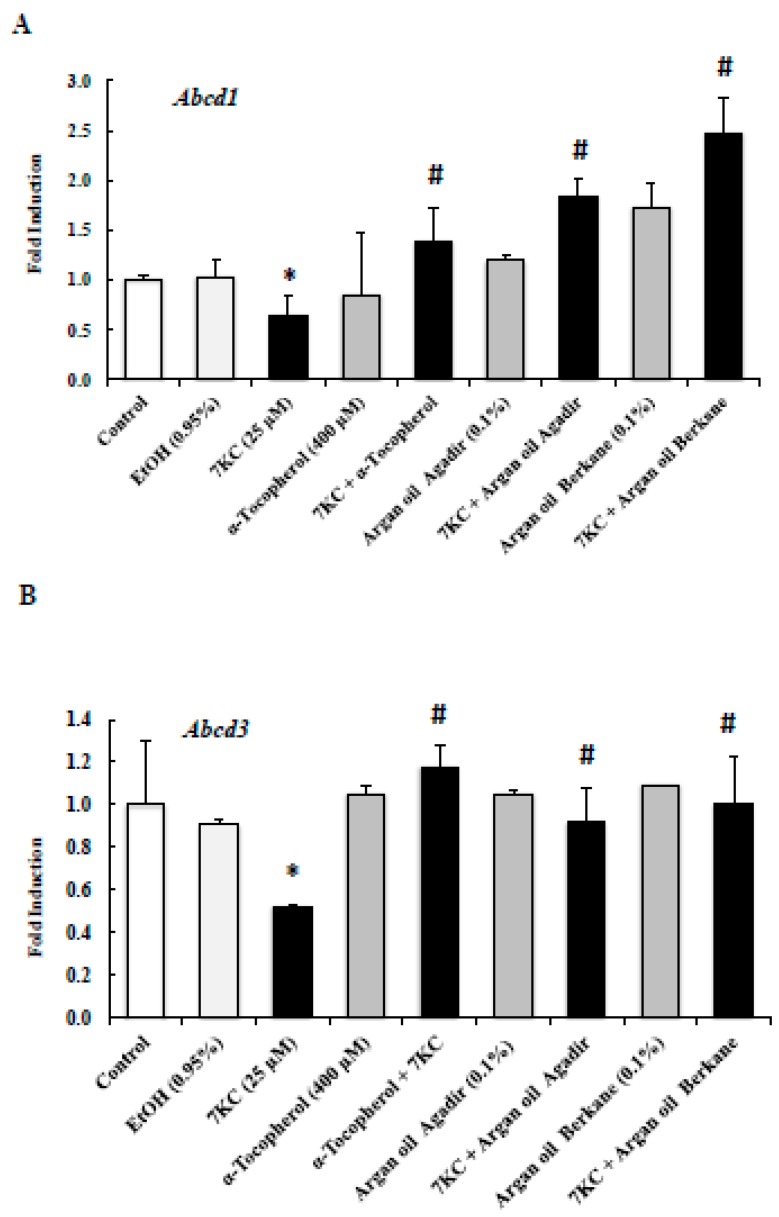

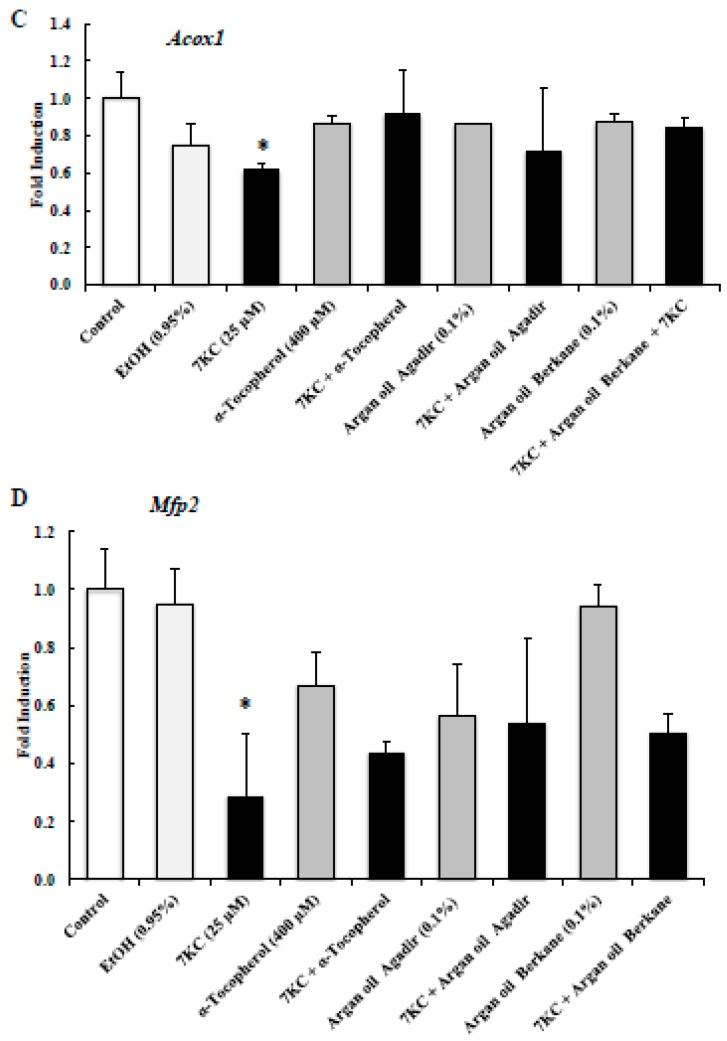
Effects of argan oil on 7-ketocholesterol-induced decreased transcription of *Abcd1*, *Acox1*, *Mfp2*, and *Abcd3*. After 24 h of culture, 158N murine oligodendrocytes were cultured for an additional 24 h without or with 7KC (25 µM) in the absence or presence of argan oils (Agadir or Berkane; Morocco; 0.1% *v*/*v*) or of α-tocopherol (400 µM) used as the positive control. Argan oils and α-tocopherol were added to the culture medium 2 h before 7KC. The mRNAs of the peroxisomal transporters (*Abcd1*) (**A**), and of the peroxisomal enzymes (*Acox1*, and *Mfp2*) (**C–D**) as well as of *Abcd3* (a marker of the peroxisomal mass); (**B**) were quantified by RT-qPCR. To this end, 36B4 was used as the reference gene. Data are mean ± SD of two independent experiments carried out in triplicate. The significance of the relationship between vehicle and cells treated with 7KC, argan oils or α-tocopherol was calculated by the Anova test (Sidak’s multiple comparisons); * *p* ≤ 0.05. The significance of the relationship between cells treated with 7KC alone, 7KC and argan oils cotreatment, or 7KC and α-tocopherol cotreatment was calculated by the Anova test (Sidak’s multiple comparisons); ^#^ *p* ≤ 0.05. No significant difference was found between control and vehicle-treated cells (EtOH 0.95% *v*/*v*).

Abcd1 (ATP binding cassette subfamily D member 1) is a major peroxisomal protein involved in the transport of very long chain fatty acids from the cytosol into peroxisomes for their degradation by β-oxidation; Acox1 (Acyl CoA oxidase 1) and Mfp2 (Multifunctional protein 2) are central oxidases of the β-oxidation pathway [7]. Abcd1, Acox1, and Mfp2 contribute to myelination and axonal integrity in the brain, and their deficiency is associated with neurodegeneration [61]. It was therefore important to determine the effects of 7KC (25 µM, 24 h) associated or not with argan oils or α-tocopherol (400 µM) on the corresponding mRNA levels of these proteins (Figure 5). The Ct values of the peroxisomal proteins associated with peroxisomal β-oxidation and of the reference gene were as follows in untreated 158N cells: *Abcd1*: 26.9 ± 0.8; *Abcd3*: 30.9 ± 0.5; *Acox1*: 30.9 ± 0.5; *Mfp2*: 28.7 ± 1.2; *36B4*: 17.6 ± 1.0, and were in the range of those previously obtained [39]. It is noteworthy that the mRNA levels of *Abcd1*, *Acox1* and *Mfp2* were strongly affected in 158N cells treated with 7KC (25 µM) (Figure 5). Interestingly, the decrease in mRNA levels of *Abcd1*, and *Acox1* induced by treatment with 7KC (25 µM) was significantly attenuated by argan oils and α-tocopherol (Figure 5). However, the decrease in the *Mfp2* mRNA level induced by 7KC was not significantly counteracted by argan oils or by α-tocopherol (Figure 5). In addition, as Abcd3 (ATP binding cassette subfamily D member 3) protein is considered a suitable marker of peroxisomal mass [62], its mRNA level was also quantified. Following treatment with 7KC, a significant decrease in the *Abcd3* mRNA level was observed, and this decrease was prevented by argan oils and α-tocopherol (Figure 5). Similar *Abcd1, Abcd3, Acox1 and Mfp2* mRNA levels were found in control and vehicle-treated cells (Ethanol (EtOH): 0.9%) (Figure 5). Compared with control and vehicle-treated cells, treatment with argan oils or α-tocopherol had no effects on *Abcd1, Abcd3, Acox1* and *Mfp2* mRNA levels.

### 2.8. Evaluation of the Effects of Argan Oils on 7KC-Induced Decreased Transcription of PPARα mRNA Level

There is now substantial evidence that peroxisome proliferator-activated receptors (PPARs), including PPAR-α, play important roles in the downregulation of mitochondrial dysfunction, proteasomal dysfunction, oxidative stress, and neuroinflammation, which are the major causes of the pathogenesis of neurodegenerative disorders [63,64,65].

**Figure 6:**
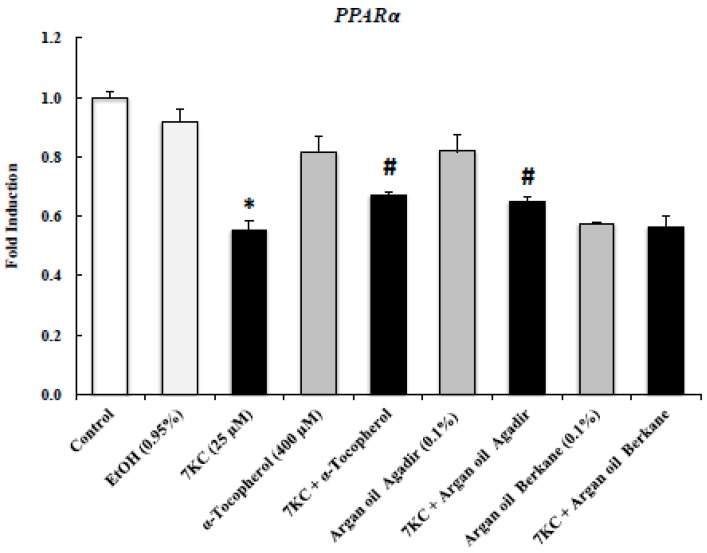
Effects of argan oil on 7-ketocholesterol-induced decreased transcription of *PPARα*. After 24 h of culture, 158N murine oligodendrocytes were cultured for an additional 24 h without or with 7KC (25 µM) in the absence or presence of argan oils (Agadir or Berkane; Morocco; 0.1% *v*/*v*) or of α-tocopherol (400 µM) used as the positive control. Argan oils and α-tocopherol were added to the culture medium 2 h before 7KC. The mRNAs of *PPARα* was quantified by RT-qPCR. *36B4* was used as the reference gene. Data are mean ± SD of two independent experiments carried out in triplicate. The significance of the relationship between vehicle and cells treated with 7KC, argan oils or α-tocopherol was calculated by the Anova test (Sidak’s multiple comparisons); * *p* ≤ 0.05. The significance of the relationship between cells treated with 7KC alone, 7KC and argan oils cotreatment, or 7KC and α-tocopherol cotreatment was calculated by the Anova test (Sidak’s multiple comparisons); ^#^ *p* ≤ 0.05. No difference was observed between control and vehicle (EtOH 0.95%)-treated cells.

It was therefore important to determine the effects of 7KC (25 µM, 24 h) alone, cotreatment with 7KC and argan oils, and cotreatment with 7KC and α-tocopherol (400 µM) on the *PPARα* mRNA level (Figure 6). Interestingly, the decrease in mRNA levels of *PPARα* induced by treatment with 7KC (25 µM) was significantly attenuated by argan oil from Agadir (but not from Berkane) and α-tocopherol but the level remained lower than that in control and vehicle-treated cells (Figure 6). Similar *PPARα* mRNA levels were found in control cells (*C_t_* value: 27.9 ± 0.3) and vehicle-treated cells (Ethanol (EtOH): 0.9%) (Figure 6). Levels of *PPARα* in cells treated with argan oils or α-tocopherol were lower than those in control and vehicle-treated cells (Figure 6).

### 2.9. Evaluation of the Effects of Argan Oils on 7KC-Induced Apoptosis and Autophagy

The effects of argan oils (0.1% *v*/v) and α-tocopherol (400 µM) on 7KC (25–50 µM, 24 h)-induced apoptosis and autophagy were evaluated by the morphological aspect of the cells after Hoechst staining (percent of apoptotic cells: cells with condensed and/or fragmented nuclei) as well as by caspase-3 activation for apoptosis, and by conversion of LC3 (light chain 3)-I into LC3-II leading to an increased (LC3-II/LC3-I) ratio for autophagy.

**Figure 7:**
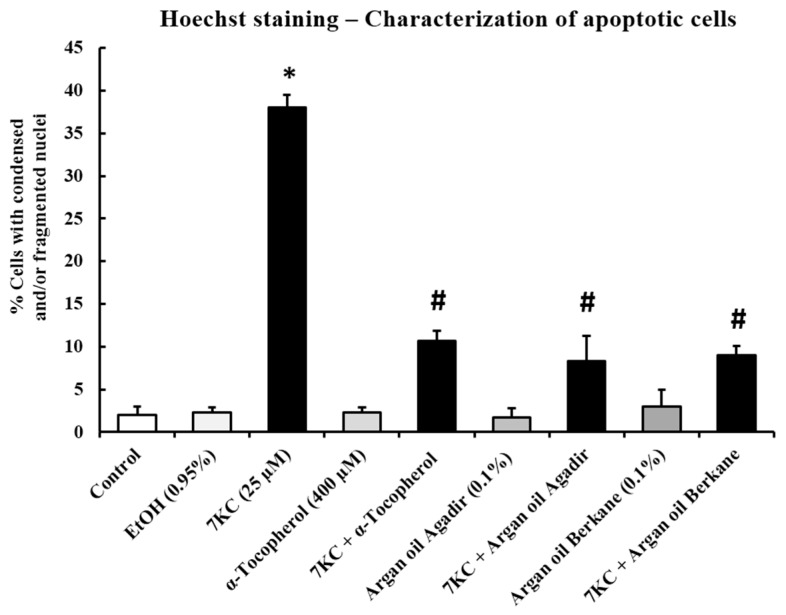
Effects of argan oil on 7-ketocholesterol-induced apoptosis evaluated by condensation and/or fragmentation of the nuclei. After 24 h of culture, 158N murine oligodendrocytes were cultured for an additional 24 h without or with 7KC (25–50 µM) in the absence or presence of argan oils (Agadir or Berkane; Morocco; 0.1% *v*/*v*) or of α-tocopherol (400 µM) used as the positive control. Argan oils and α-tocopherol were added to the culture medium 2 h before 7KC. Apoptosis was evaluated by the percentage of apoptotic cells characterized by condensed and/or fragmented nuclei whereas control cells (untreated cells) have round and regular nuclei. Data are mean ± SD of two independent experiments carried out in triplicate. The significance of the relationship between vehicle and cells treated with 7KC, argan oils or α-tocopherol was calculated by the Anova test (Sidak’s multiple comparisons); * *p* ≤ 0.05. The significance of the relationship between cells treated with 7KC alone, 7KC and argan oils cotreatment, or 7KC and α-tocopherol cotreatment was calculated by the Anova test (Sidak’s multiple comparisons); ^#^ *p* ≤ 0.05. No difference was observed between control and vehicle (EtOH 0.95%)-treated cells.

**Figure 8:**
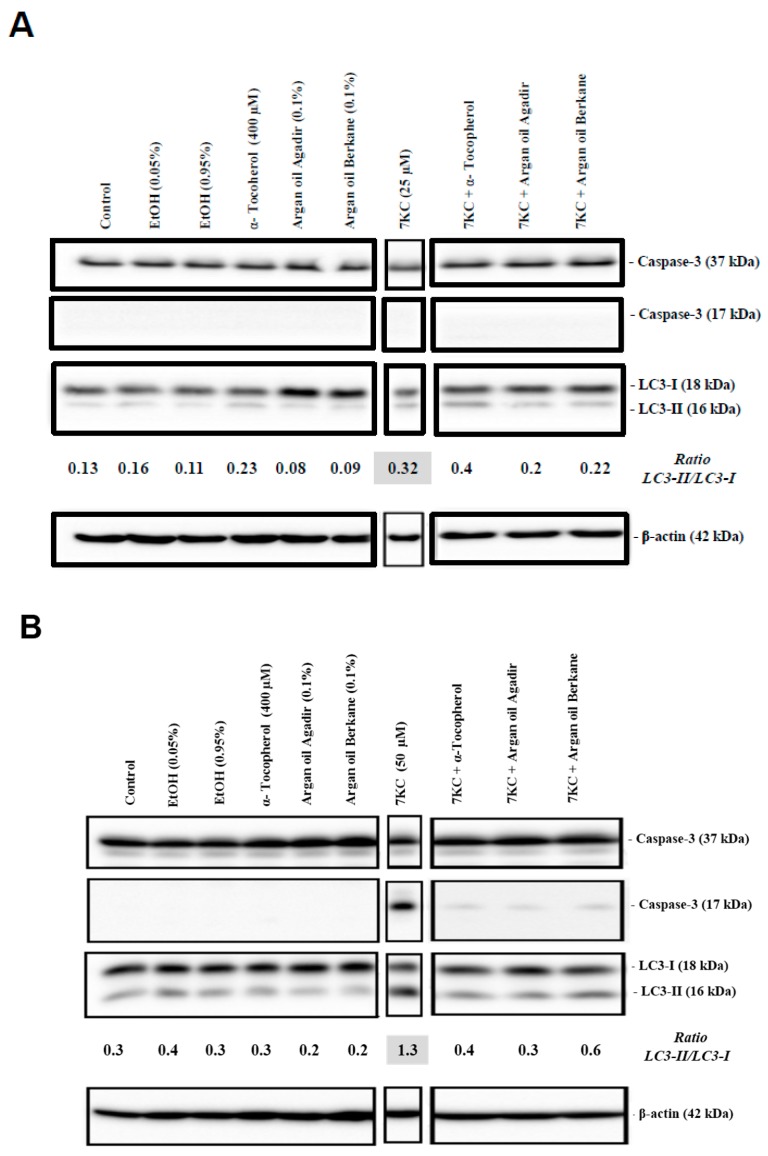
Effects of argan oil on 7-ketocholesterol-induced cleaved caspase-3 and activation of LC3-I into LC3-II. After 24 h of culture, 158N murine oligodendrocytes were cultured for an additional 24 h without or with 7KC (25 µM) (**A**) or with 7KC (50 µM) (**B**) in the absence or presence of argan oils (Agadir or Berkane; Morocco; 0.1% *v*/*v*) or of α-tocopherol (400 µM) used as the positive control. Argan oils and α-tocopherol were added to the culture medium 2 h before 7KC. Apoptosis was evaluated by caspase-3 activation (cleaved caspase-3) and autophagy by conversion of LC3-I to LC3-II (increased LC3-II/LC3-I ratio). The EtOH values (0.05%, 0.1% and 0.9% *v*/*v*) correspond to the final EtOH concentration in the culture medium with 7KC (25 µM), 7KC (50 µM) and argan oils, respectively. No difference was observed between control and vehicle (EtOH)-treated cells. Data shown are representative of three independent experiments.

As shown by fluorescence microscopy and by Western blotting, in the presence of 7KC (25 and 50 µM; 24 h), both argan oils (0.1% *v*/*v*) and α-tocopherol (400 µM) were able to counteract: (i) apoptosis revealed by a moderated increase in the percentage of apoptotic cells with 7KC (25 µM) (7KC (25 µM): 15 ± 2%; control: 2 ± 1%; vehicle (EtOH): 3 ± 1%) and by a marked increase in the percentage of apoptotic cells associated with cleaved caspase-3 with 7KC (50 µM) ((7KC (50 µM): 42 ± 3%; control: 2 ± 1%; vehicle (EtOH): 3 ± 1%)); and (ii) autophagy revealed by the activation of LC3-I into LC3-II with 7KC (25 and 50 µM) (Figure 7 and Figure 8). In the presence of 7KC (25 µM, 24 h), the absence of cleaved caspase-3 (Figure 8A) could be due to the low percentage of apoptotic cells, and to the limit of detection. However, with a low concentration of 7KC (25 µM), autophagy was detected and the activation of LC3-I into LC3-II was revealed. This activation was attenuated by argan oils but not by α-tocopherol (Figure 8A). When 158N cells were treated with vehicle (EtOH 0.9% *v*/*v*), argan oils (0.1% *v*/*v*) or α-tocopherol (400 µM), no cleaved caspase-3 was observed (Figure 8A).

Following treatment with 7KC (50 µM, 24 h), and in agreement with previous studies [6,55,60,66,67], the percentage of apoptotic cells was markedly increased (Figure 7), and the activation of caspase-3 revealed by the presence of cleaved caspase-3 and by the activation of LC3-I into LC3-II revealed by an increase in the (LC3-II/LC3-I) ratio was observed (Figure 8B). Thus, at a high concentration, 7KC (50 µM) was able to trigger both autophagy and apoptosis. Of note, the activation of caspase-3 and of LC3-I into LC3-II was strongly attenuated by argan oils (0.1% *v*/*v*) and α-tocopherol (400 µM). When 158N cells were treated with vehicle (EtOH 0.9% *v*/*v*), argan oils (0.1% *v*/*v*) or α-tocopherol (400 µM), no increase in the percentage of apoptotic cells was found in comparison with controls and no caspase-3 cleavage was observed (Figure 7 and Figure 8B).

## 3. Discussion

Dietary habits are now considered major parameters in the development of numerous age-related diseases. The first evidence of the effects of diet on the onset of cardiovascular diseases appeared in the second half of the 20th century. In this context, Jean Renaud introduced the notion of the “French paradox”, according to which the moderate consumption of red wine has beneficial effects for the prevention of cardiovascular diseases [68,69]. This led to the identification of polyphenols as molecules with cardioprotective properties [70]. From another viewpoint, as polyphenols are contained in olive oil and certain vegetables and fruits associated with the Mediterranean diet, which is considered beneficial for human health, especially the prevention of cardiovascular disease [71,72], consumers have become more and more aware of the importance of diet on health. Currently, given the high number of patients with age-related neurodegenerative diseases (especially dementia) worldwide, it has become important to know the beneficial and detrimental effects of food and nutrients on the development of neurodegenerative diseases. At the moment, concerning the development of dementia, beneficial and detrimental effects have been attributed to omega-3 and *trans*-fatty acids, respectively [9,73]. Polyphenols contained in olives and wine seem to be promising to combat aging-related neurodegeneration [10,74]. It is also supposed that the dietary intake of phytosterols might be beneficial in preventing Alzheimer’s disease [75,76]. Additionally, tocopherols have been reported to influence different mechanisms involved in the pathogenesis of Alzheimer’s disease, e.g., Aβ-aggregation, Aβ-induced neurotoxicity, oxidative stress, and inflammatory processes [77]. Therefore, the regular consumption of oils containing fatty acids, phytosterols, polyphenols and tocopherols, which are able to pass the blood-brain barrier, might contribute to preventing neurodegeneration. In this context and as few data are currently available [20,29,30,78], it was of interest to establish the profiles of argan oils from Agadir and Berkane (two main producing area of argan oil in Morocco) with regard to fatty acids, phytosterols, tocopherols and polyphenols, since these compounds are potentially neuroprotective, and to determine their anti-oxidant characteristics with in vitro assays (KRL and FRAP) as well as their cytoprotective effects on nerve cells.

It is widely accepted that oxidative stress and mitochondrial dysfunctions are key events in several degenerative diseases [79]. There are also several indications that the peroxisome is involved in ageing processes [80,81] and in the pathophysiology of neurodegenerative diseases [23,82]. This is clearly established in peroxysomopathies associated with peroxisomal deficiencies or dysfunctions [83], and there is also some evidence of these mechanisms in multiple sclerosis [62,84] and Alzheimer’s disease [85,86,87]. Furthermore, in autophagy, which is considered beneficial to prevent neurodegeneration, the lysosome is involved and fused with the autophagosome to form the autophagolysosome [88].

Interestingly, the profile of argan oils from Agadir and Berkan reveal the presence of numerous compounds able to prevent oxidative stress and to counteract mitochondrial, lysosomal, and peroxisomal dysfunctions in stress conditions. For example, the argan oils studied, as well as extra virgin olive oil from Tunisia, contain high levels of anti-oxidant molecules (tocopherols, polyphenols) able to impair the overproduction of reactive oxygen species (ROS: ROO^●^, RO^●^) [89], which can induce lipid peroxidation subsequently leading to protein carbonylation and to several cellular dysfunctions that can contribute to neurodegeneration [90]. The presence of these compounds is in keeping with the antioxidant properties of argan and extra virgin olive oils determined with the KRL and FRAP assays. In addition, argan and extra virgin olive oils are also rich in oleic acid (C18:1 n-9). Although this fatty acid is not an anti-oxidant [35], it has been clearly established in murine microglial BV-2 cells treated with 7KC that (C18:1 n-9) was able to attenuate 7KC-induced ROS overproduction [35,54]. In addition, oleic acid, and α- and γ-tocopherol have been shown to prevent mitochondrial, lysosomal and peroxisomal dysfunctions in different types of nerve cells from different species [35,39,54,66]. As these compounds, which have been shown to prevent 7KC-induced neurotoxicity in vitro, are present at low levels in argan and olive oils (the final concentrations of oleic acid, α- and γ-tocopherol were more than a hundred times lower than those in the culture medium when these compounds were used alone) [35], it is possible that some of them could act synergistically to exert cytoprotective effects, and/or that several other compounds of argan oils (polyphenolic compounds, stilbenes, etc.) [91,92] could be involved in these beneficial effects. Thus, from a chemical point of view, the cytoprotection observed with the oils may be linked to the antioxidant potential of the mixture of molecules, which may be amplified in comparison with the molecules used alone. From a biological point of view, we also have to consider that the different compounds present are simultaneously able to activate and repress several signaling pathways which could contribute to cytoprotection.

Of note, when 7KC was used at 25 and 50 µM, two concentrations able to induce oxiapoptophagy (OXIdation + APOPTOsis + autoPHAGY) [59,60,93], argan was able to attenuate 7KC (25–50 µM)-induced toxicity. As observed for α- and γ-tocopherol [35], docosahexahenoic acid [60], oleic acid [35] and dimethylfumarate [58], argan oil (0.1% *v*/*v*) was able to attenuate 7KC-induced inhibition of cell growth associated with a loss of cell adhesion. These results were revealed by phase-contrast microscopy and the crystal violet and MTT test. As observed with α- and γ-tocopherol [35], docosahexahenoic acid [60], oleic acid [35], polyphenols [94,95,96,97] and dimethylfumarate [58], argan oil was also able to markedly reduce oxidative stress, evaluated as the percentage of cells overproducing superoxide anions revealed by staining with DHE, and the percentage of cells permeable to PI, which can correspond to dead cells and/or to cells with damaged plasma membranes, as a consequence, at least in part, of lipid peroxidation and of the disorganization of the lipid membrane occurring as a result of treatment with 7KC [54,98]. The cytoprotective effect of argan oil on the plasma membrane is in agreement with previous data from studies on retinal pigment epithelial (RPE) cells, which showed that argan oil is incorporated into retinal cells and increased plasma membrane fluidity [99]. In addition, 7KC-induced organelle dysfunctions were also counteracted by argan oil: mitochondrial hyperpolarization revealed with the MTT test was attenuated, as was the increase in numbers of acidic vesicles (regarded as lysosomes) and/or the increase in their size, as revealed by staining with AO, probably related to 7KC-induced autophagy [60,100]; peroxisomal alterations revealed by lower mRNA levels of *Abcd1*, *Abcd2*, *Acox1* and *Mfp2*, which encode for transporters or enzymes involved in peroxisomal β-oxidation [7] were also attenuated as was the mRNA level of *PPARα*, a nuclear transcription factor involved in controlling the number and size of peroxisomes, especially in mice, and which also plays important roles in the downregulation of mitochondrial dysfunction, proteasomal dysfunction, oxidative stress, and neuroinflammation [63,64,65]. It was previously reported that two phytosterols, schottenol and spinasterol, which are considered specific to argan oil, as well as sterol extracts from argan oil were not toxic to microglial murine BV-2 cells, even though spinasterol and schottenol were able to modulate mitochondrial membrane potential and the gene expression of two nuclear receptors, liver X receptor (LXR)-α and LXR-β, as well as their target genes *ABCA1* and *ABCG1* [101]. In addition, in agreement with the ability of argan oil to affect peroxisome biogenesis and/or functions, it was also shown that argan oil induced peroxisome proliferation in fibroblasts from patients with pseudo-neonatal adrenoleukodystrophy (P-NALD/ACOX1 deficiency), and that this induction was independent of the activation of both nuclear receptor PPAR*α* and its coactivator PGC-1α [102]. In agreement with our data, it was also previously reported that argan oil was able to normalize *PPARα* mRNA levels in stress conditions. For example, when argan oil was incorporated into standard chow, it was able to normalize the hepatic mRNA expression of the nuclear receptors *PPARα* in mice treated with purified endotoxin (lipopolysaccharide) [103]. In addition, argan oil was able to prevent 7KC-induced activation of LC3-I into LC3-II, and the cleavage of caspase-3, which are criteria of autophagy and apoptosis, respectively. In the presence of 7KC used at 25 μM, the activation of LC3 was demonstrated by Western blotting, even though no caspase-3 cleavage was observed (despite a small increase in the percentage of apoptotic cells), suggesting that autophagy may precede apoptosis, and that this order may prevent mitochondrial and peroxisomal abnormalities. This hypothesis is in agreement with the mitochondrial and peroxisomal dysfunctions revealed by the MTT and RT-qPCR tests, respectively, as well as the increase in the percentage of positive AO cells. In contrast, the autophagy induced by 7KC used at 50 μM could be deleterious, and the release of proteolytic enzyme by the autophagolysosome could amplify cell death and contribute to further apoptosis [104]. Currently, the autophagic process associated with 7KC-induced cell death is considered rather beneficial [105]. These beneficial and deleterious aspects of autophagy are well documented, particularly in neurodegenerative diseases [106]. The fact that some compounds of argan oil can act on this process, as well as on the apoptosis also involved in neurodegeneration, is an important argument in favor of the potentially neuroprotective activity of argan oil.

Since argan oils contain numerous compounds, we can wonder whether some of them could act as Pan Assay Interference Compounds (PAINS) [107]. Indeed, PAINS include numerous types of molecules from different structures which can interfere with readouts in multiple ways (protein reactivity, chelation, redox activity, fluorescence induction, etc.) [108], and could therefore lead, if not to artifactual data, at least to non-specific data. This is especially relevant for polyphenols [109]. However, as polyphenols are mainly hydrophilic molecules present at very low levels in oils, their cytoprotective activity on 7KC-induced oxiapoptophagy can reasonably be excluded. On the other hand, as fatty acids (especially oleic acid) and tocopherols (mainly alpha- and gamma-tocopherol) are present at elevated levels in argan oil, and have previously been shown to prevent 7KC-induced oxiapoptophagy in both microglial BV-2 cells and 158N cells [35,39,58], we rather supposed that these compounds contribute to the cytoprotective effects of argan oil. As several data are available on the signaling pathways activated by 7KC [48,55,98,110] and on the cell targets of tocopherols and oleic acid involved in the attenuation of 7KC-induced side effects [35,54,66,110], it will be easy to determine whether this hypothesis is realistic. The data obtained in the present work (attenuation of organelle dysfunction and oxiapoptophagy) support this hypothesis. However, to exclude the involvement of PAINS, it will be useful to determine whether argan oil contains PAINS structures and to measure their concentrations [109]. It remains to define how PAINS alerts must be used in order to identify PAINS with accuracy [111].

In conclusion, the present study provides new data reinforcing the interest of the use of argan oil for the prevention of neurodegenerative diseases and supports the importance of dietary habits to prevent neurodegeneration. The chemical profile of argan oil, which revealed the presence of fatty acids, polyphenols, phytosterols and tocopherols able to attenuate various side effects associated with neurodegeneration (oxidative stress, organelle dysfunctions, and cell death), reinforces the interest of argan oil to prevent neurodegenerative diseases. In addition, argan oil has anti-oxidant properties, and is also able to counteract 7KC-induced oxiapoptophagy in 158N murine oligodendrocytes. As 7KC is often increased in the plasma, cerebrospinal fluid and brain of patients with neurodegenerative diseases, the cytoprotective effects of argan oil against the toxic effects of 7KC suggest that argan oil may have beneficial effects in preventing or slowing the development of some neurodegenerative diseases. Altogether, the results obtained on the cytoprotective effects of argan oil on murine 158N oligodendrocytes encourage us to use more elaborate cell models that take into account the selective passage of certain compounds of argan oil through the blood–brain barrier. These models, which mimic the blood-brain barrier by combining cell cultures (endothelial cells, pericytes and nerve cells) [112,113], should provide a more precise idea of the activity of argan oil on the cells of the central nervous system (glial and microglial cells, neurons) under normal conditions or under stress conditions induced by different agents.

## 4. Experimental Section

### 4.1. Cell Culture and Treatments

Murine oligodendrocytes (158N) [114] were seeded at 30,000 cells/cm^2^ either in Petri dishes (100 × 20 mm, FALCON, Corning, NY, USA) with 10 mL of culture medium, or at 240,000 cells per well on six-well plates with 2 mL of culture medium. They were cultured in Dulbecco’s Modified Eagle Medium (DMEM) (Lonza, Amboise, France) supplemented with 5% (*v*/*v*) heat-inactivated fetal bovine serum (FCS) (Dutscher, Brumath, France) and 1% antibiotics (penicillin and streptomycin) (Dutscher). The cells were incubated at 37 °C in a humidified atmosphere containing 5% CO_2_. For subcultures, cells were trypsinized (0.05% trypsin-0.02% EDTA solution, (Dutscher)), and passaged twice a week.

7KC (Ref: C2394) was from Sigma-Aldrich (St Quentin Fallavier, France). The stock solution of 7KC was prepared at 800 µg/mL (2 mM) as previously described [66]. After 24 h of culture, 158N cells were incubated for an additional 24 h without or with 7KC (25 and 50 µM) in the absence or presence of argan oil or α-tocopherol (Sigma-Aldrich: 400 µM) used as the positive control to attenuate the toxic effects of 7KC [66]. 7KC concentrations (25 and 50 µM)) and the time of treatment (24 h) were chosen because they are known to induce mitochondrial, peroxisomal and lysosomal dysfunctions [39,55,115].

A stock solution of argan oil (dietary argan oil from Agadir or Berkane) was prepared at 10% (*v*/*v*) in absolute ethanol. For cell treatment, one volume of this stock solution was introduced in one hundred volumes of culture medium: the final concentration of oil was 0.1% (*v*/*v*), and the final corresponding ethanol concentration was 0.9%. These dilution conditions made it possible to solubilize a maximum of oil by introducing a final quantity of ethanol into the culture medium without affecting the cellular activity (RedOx status, organelle activity, cell viability) [115]. When the cells were treated with 7KC associated with argan oil or with α-tocopherol (Sigma-Aldrich), these compounds were added 2 h before the 7KC (supplementary Figure S1).

The concentrations used for 7KC and α-tocopherol were chosen for the following reasons. In vitro, the cytotoxic effect of 7KC is evaluated by its 50% inhibiting concentration (IC50), which is in the range of 25–50 µM in different cell types [116]. These concentrations commonly used in toxicological studies permit comparisons from one cell type to another, and it is important to underline that only 10–35% of 7KC introduced into the culture medium accumulates within the cells [117,118]. Interestingly, similar observations were made with 27-hydroxycholesterol [119]. Based on these considerations, it can be supposed that the intracellular oxysterol content obtained in vitro could be in the range of those occurring in vivo. Furthermore, it is important to emphasize that the use of 7KC (25–50 µM) in different cell types is a relevant model: (i) to evaluate the relationship between oxidative stress, apoptosis and autophagy; (ii) to specify the role played by mitochondria, peroxisomes and lysosomes in these processes; (iii) to determine the interactions between mitochondria and peroxisome in 7KC-induced lipotoxicity; and (iv) to identify natural and synthetic molecules (or mixture of molecules such as oils) to prevent the toxic effects of 7KC associated with major age-related diseases and with several neurodegenerative diseases [39,40,41,42]. α-tocopherol, the major compound of Vitamin E, was used at the highest non-cytotoxic concentration (400 µM) able to prevent 7KC-induced oxiapoptophagy (oxidative stress, apoptosis and autophagy) in numerous cell types, including nerve cells [39,54,60,66]. With α-tocopherol, it has previously been reported that the strongest cytoprotective effects in 158N cells were observed at 200 and 400 µM [66]. Therefore, in the present study, α-tocopherol was used at 400 µM, the most efficient concentration able to prevent 7KC-induced side effects.

### 4.2. Determination of the Fatty Acid Profile of Argan Oil *Versus* Extra Virgin Olive Oil by Gas Chromatography

Dietary argan oils were from Morocco (Berkane, Agadir) and obtained by mechanical extraction. Currently, the extraction of the argan oil is carried out either manually (artisanal method) or mechanically (industrial method) [20,22,120]. It is done in five stages: pulping the fruit, crushing the hull, roasting the almond, grinding roasted almonds either using a stone wheel and manual pressure, or using a mechanical press. Extra virgin olive oil was from Tunisia (Mahdia). It was obtained from artisanal manufacturers and simply made by crushing olives and extracting the juice. The oils were stored at 4 °C until analysis, and were analyzed between 6–12 months after being obtained. Lipids were extracted from the different oils according to the Moilanen and Nikkari method [121]. C19:0 was used as the internal standard. Lipids were transmethylated using boron trifluoride in methanol according to Morrison and Smith [122]. Fatty acid methyl esters were subsequently extracted with hexane and analyzed using gas chromatography on a Hewlett Packard Model 5890 gas chromatograph (Palo Alto, CA, USA) using a CPSIL-88 column (100 m × 0.25 mm i.d., film thickness 0.20 µm; Varian, Les Ulis, France) equipped with a flame ionization detector (FID). Hydrogen was used as the carrier gas (inlet pressure, 210 kPa). The oven temperature was held at 60 °C for 5 min, increased to 165 °C at 15 °C/min and held for 1 min, and then to 225 °C at 2 °C/min and finally held at 250 °C for 17 min. The injector and the detector were maintained at 250 °C and the samples were injected. Fatty acid methyl esters were identified by comparison with commercial and synthetic standards (Sigma Aldrich). The data were processed using EZChrom Elite software (Agilent Technologies, Massy, France) and reported as mg/g of total lipids.

### 4.3. Determination of the Tocopherol Profile of Argan Oil versus Extra Virgin Olive Oil by High Pressure Liquid Chromatography

Forty milligrams of argan oil (Berkane and Agadir, Morocco) or extra virgin olive oil (Mahdia, Tunisia) was resuspended in 1 mL of a mixed high performance liquid chromatography (HPLC) mobile phase: acetonitrile/methanol containing 50 mM ammonium acetate/water/dichloromethane (700:150:50:100, *v*/*v*/*v*/*v*). After resuspension, the extract was vortexed for 30 s. Samples of 80 µL were injected into the HPLC system for the analysis of Tocopherols. The analytical conditions were based on those reported by Lyan et al. [123]. The HPLC apparatus was a Jasco PU-1580 Plus intelligent pump equipped with an automatic injector system AS300 (Thermo Finnigan, Les Ulis, France) and a Jasco MD-1510 plus multiwavelength detector (JASCO International Co., Ltd., Tokyo, Japan). HPLC analyses were carried out using RPHPLC with a Nucleosil C18 column (250 mm × 4.6 mm internal diameter (id), 5 µm particle size) and a VIDAK C18 column (250 mm × 4.6 mm id, 5 µm particle size) under isocratic conditions. The mobile phase consisted of a mixture of acetonitrile/methanol at 50 mM ammonium acetate/water/ dichloromethane (700:150:50:100, *v*/*v*/*v*/*v*), at a flow rate of 2 mL/min. The tocopherols were detected at 298 nm, their identification was ensured by comparing the retention times and absorption spectra with reference standards and their quantification was ensured using standard curves for each compound. Six quantities of α-tocopherol (range: 0.5–8.5 µg), γ-tocopherol (range: 1–25 µg) and δ-tocopherol (range: 0.1–2 µg) were injected into the HPLC system (each standard being dissolved in 1 mL of the HPLC mobile phase: acetonitrile/methanol containing 50 mM ammonium acetate/water/dichloromethane (700:150:50:100, *v*/*v*/*v*/*v*); the linear regression equation for each standard curve was then obtained by plotting the amount of the standard compound injected against the peak surface area. The regression equation and correlation coefficient (r^2^) were calculated using ChromNav software (JASCO).

### 4.4. Extraction by Organic Solvent

#### 4.4.1. Plant Material

The almonds obtained from argan nuts of Argania spinosa used in this study also came from Berkane and Agadir (Morocco). The argan nuts were harvested between February and April 2014. Freshly harvested, the almonds were extracted, washed with distilled water and dried overnight in an oven at 50 °C under ventilation to preserve the integrity of their chemical composition. After drying, the almonds were finely ground with a grinder (SODIPRO, Grenoble, France) and then screened on sieves.

#### 4.4.2. Preparation of Crude, Lipidic and Non-Lipidic Extracts

The preparations of crude, lipidic and non-lipidic extracts were based on the method described by Mahmoudi et al. [124]. To this end, 10 g of almond powder was macerated in 75 mL of absolute methanol overnight (12 h) with gentle stirring. The macerate was filtered on Wattman paper grade 4. The filtrate was evaporated under vacuum using a rotary rotavapor (BUCHI, Rungis, France), at a temperature of 45 °C, to obtain a crude extract containing all secondary metabolites. A part of this extract (1 mL) was mixed with hexane (*v*/*v*), and after settling and decantation (12 h, 4 °C), the recovered upper organic phase was considered the lipid fraction. The corresponding hexane extract was considered the lipid extract (LipE). The remainder of this fraction was considered the non-lipid extract (Non-LipE).

### 4.5. KRL Test

The antioxidant potential of argan and extra virgin olive oils, and of α-tocopherol was evaluated with the KRL (Kit Radicaux Libres) test [35,60,125]. Briefly, the red blood cells of defibrinated horse blood (Biomérieux, France) were oxidized by molecular oxygen in an aqueous suspension using the azo-compound 2-2′-azo-bis-(2-amidinopropane) hydrochloride (AAPH) as the free radical initiator without or with α-tocopherol, and Trolox used at (5, 10, 20, 50 and 100 µM). Several parameters were calculated from the time-dependent curve of AAPH-induced hemolysis. The time required to achieve 50% hemolysis measured by the optical density of hemoglobin was determined (red blood cell half-hemolysis time in min). Data are presented as Trolox equivalents. Hemolysis was recorded using a 96-well microplate reader by measuring the optical density decay at 450 nm (Sunrise spectrophotometer, Tecan, Männedorf, Switzerland). For each well, absorbance measurements were performed 75 times, once every 150 s.

### 4.6. Ferric Reducing Antioxidant Power (FRAP) Assay

The ferric reducing antioxidant power (FRAP) assay measures the antioxidant potential of compounds or of mixtures of compounds through the reduction of ferric iron (Fe^3+^) to ferrous iron (Fe^2+^) [126,127]. This method was used to compare the antioxidant potential of organic extracts of argan almonds as well as argan and extra virgin olive oils, and of α-tocopherol compared with Trolox (2.5–160 mg/mL corresponding to 10–640 mM) used as the reference. Briefly, for organic extracts, 1 mL of extract or Trolox was mixed with 2.5 mL of phosphate buffer solution (0.2 M; pH 6.6) and with 2.5 mL of a 1% potassium ferricyanide solution (K_3_Fe(CN)_6_; 30 mM). This mixture was then incubated in a water bath (50 °C; 20 min), and then 2.5 mL of 10% trichloroacetic acid were added to stop the reaction. The tubes were centrifuged at 1.000× *g* for 10 min. Then, 2.5 mL of the supernatant were mixed with 2.5 mL of distilled water and 0.5 mL of a 0.1% aqueous solution of ferric chloride (FeCl_3_). The absorbance of the reaction medium was read at 700 nm against a similarly prepared blank by replacing the extract with distilled water making it possible to calibrate the spectrophotometer. An increase in absorbance (Abs) corresponds to an increase in the reducing power of the extracts tested [128]. The reducing power of the extracts in mg Trolox per g dry matter (mg Trolox/g dry matter) was calculated as follows:

Iron Reducing Power (%) = [(Abs 700 Control − Abs 700 Sample)/Abs 700 Control] × 100

For argan oils, extra virgin olive oil and α-tocopherol used as the positive control, the FRAP method was carried out as previously described [35]. For the different oils analyzed, the corresponding antioxidant power was expressed as Trolox equivalent for 1 mL of oil.

### 4.7. Crystal Violet Test

Cell proliferation (evaluation of adherent cells) was estimated by staining with crystal violet (Sigma-Aldrich) in 12-well plates [129]. At the end of the treatment, cells were washed with PBS, stained with crystal violet (Sigma-Aldrich) for 5 min, and rinsed with water three times. Absorbance was read at 598 nm after extraction of the dye with 0.1 mol/L sodium citrate (Sigma-Aldrich) in 50% ethanol (Carlo-Erba, Val de Reuil, France).

### 4.8. Measurement of Mitochondrial Activity with the MTT Test

The MTT assay was used to evaluate the effects of treatments on mitochondrial activity. The MTT assay was carried out as previously described [130] on 158N cells plated in 6-well flat-bottom culture plates. MTT salt is reduced to formazan in metabolically active cells by the mitochondrial enzyme succinate dehydrogenase from NADH (Nicotinamide adenine dinucleotide) and NADPH (nicotinamide adenine dinucleotide phosphate) [131]. Succinate dehydrogenase corresponds to succinate-coenzyme Q/respiratory complex II, which is localized at the inner mitochondrial membrane and participates in both the citric acid cycle and electron transport in oxidative phosphorylation. The plates were read at 570 nm with a microplate reader (Tecan Sunrise, Tecan, Lyon, France).

### 4.9. Measurement of Acidic Vesicles with Acridine Orange

Acridine orange (AO) is a weak base which accumulates in its charged form within lysosomes of living cells because of the low lysosomal pH, and which produces red fluorescence when excited by blue light [132]. During prolonged exposure to cytotoxic agents, the red fluorescence of AO decreases markedly. The shift in AO fluorescence from granular red to diffuse green reflects leakage and redistribution of AO from the lysosomes, indicating impairment of the lysosome membranes or the inability of the lysosomes to maintain low pH [133,134,135]. The orange/red fluorescence of AO is widely used to visualize and quantify autophagic vesicles [100]. A 1 mg/mL stock solution of AO (Sigma-Aldrich) was prepared in distilled water. After staining with AO (2 µg/mL; 15 min; 37 °C), cells were washed, resuspended in PBS, and analyzed by flow cytometry. The fluorescent signals were measured on a Galaxy flow cytometer (Partec, Görlitz, Germany). The orange fluorescence of AO was collected through a 590/20 nm band pass filter. Fluorescence was quantified in 10,000 cells. Data were analyzed with Flomax (Partec) or FlowJo (Tree Star Inc., Ashland, OR, USA) software.

### 4.10. Measurement of Superoxide Anion Production with Dihydroethidium

Overproduction of superoxide anion (O_2_^●−^) was detected with dihydroethidium (DHE; Life Technologies, Carlsbad, CA, USA) [136,137]. DHE diffuses through cell membranes, and is rapidly oxidized in ethidium under the action of reactive oxygen species, mainly O_2_^●−^ [136]. DHE (1.6 mM) was prepared in dimethyl sulfoxide, and used at 2 μM. After 15 min at 37 °C, the fluorescent signals of DHE-stained cells were collected through a 590/20 nm band pass filter, on a logarithmic scale on a GALAXY flow cytometer (Partec); 10,000 cells were acquired; data were analyzed with Flomax (Partec) or FlowJo (Tree Star Inc.) software.

### 4.11. Measurement of Plasma Membrane Permeability with Propidium Iodide

158N cells were stained with 1 μg/mL of propidium iodide (PI), which enters dead cells or cells with damaged cytoplasmic membranes only [138]. Fluorescence was collected using a 630 longpass filter. Flow cytometric analyses were performed on a Galaxy flow cytometer (Dako/Partec; Münster, Germany). Ten thousand cells were acquired for each sample. Data were analyzed with Flomax software (Partec).

### 4.12. Quantification of Apoptotic Cells after Staining Nuclei with Hoechst 33342

Nuclear morphology of control and oxysterol-treated cells was characterized by fluorescence microscopy with Hoechst 33342 (2 µg/mL). Apoptotic cells were characterized by nuclear condensation of chromatin and/or nuclear fragmentation [138]. Deposits of about 40,000 cells were applied to glass slides by cytocentrifugation (5 min, 1500 rpm) with a cytospin 2 (Shandon, Cheshire, UK), mounted in Dako fluorescent mounting medium (Dako, Copenhagen, Denmark) and stored in the dark at 4 °C until observation. The morphological aspect of the cell nuclei was determined with an Axioskop fluorescent microscope (Zeiss, Oberkochen, Germany). For each sample, 300 cells were examined.

### 4.13. Quantification of Abcd1, Abcd2, Abcd3, Acox1, Mfp2 and PPARα mRNAs by RT-qPCR

Total mRNA from 158N cells was extracted and purified using the RNeasy Mini Kit (Qiagen, Hilden, Germany) with 15 min DNAse treatment (Qiagen). Total mRNA concentration was measured with TrayCell (Hellma, Paris, France) and the purity of nucleic acids was controlled by the ratio of absorbances at 260 nm and 280 nm (ratios between 1.8 and 2.2 were considered satisfactory). One microgram of total mRNA was used for reverse transcription with the iScript cDNA Synthesis Kit (Bio-Rad, Hercules, CA, USA) according to the following reaction protocol: 5 min at 25 °C, 1 h at 42 °C, 5 min at 85 °C. cDNA was amplified using the MESA GREEN qPCR MasterMix Plus for SYBR Assay w/fluorescein (Eurogentec, Liège, Belgium). All PCR reactions were performed on an Applied Biosystem Step One plus QPCR machine (Life Science Technologies / Thermo Fisher scientific, Villebon sur Yvette, France). The primer sequences were the followings:

*Abcd1:* forward 5′-gccaagttgtggatgag-3′ and reverse 5′-ttccgcagagtcgggataga-3′
*Abcd2:* forward 5′-tagaccgcatcctgcacagc-3′ and reverse 5′-ctccttcgccatcgaattgt-3′
*Abcd3:* forward 5′-ctgggcgtgaaatgactagattg-3′ and reverse 5′-cttctcctgttgtgacaccattg-3′
*Acox1:* forward 5′-gcccaactgtgacttccatt-3′ and reverse 5′-ggcatgtaacccgtagcact-3′
*Mfp2:* forward 5′-aggggacttcaagggaattgg-3′ and reverse 5′-gcctgcttcaactgaatcgtaa-3′
*PPARα:* forward 5′-tattcggctgaagctggtgtac-3′ and reverse 5′-ctggcatttgttccggttct-3′

Thermal cycling conditions were as follows: activation of DNA polymerase at 95 °C for 10 min, followed by 40 cycles of amplification at 95 °C for 15 s, 60 °C for 30 s, and 72 °C for 30 s, followed by a melting curve analysis to control for the absence of non-specific products. Gene expression was quantified using cycle to threshold (*C*_t_) values and normalized by the *36B4* reference gene (forward 5′-atctgcttggagcccacat-3′ and reverse 5′-gcgacctggaagtccaacta-3′). The quantitative expression of *Abcd1*, *Abcd2*, *Abcd3*, *Acox1*, *Mfp2* and *PPARα* was determined as fold induction of the control.

### 4.14. Analysis of Caspase-3 and LC3 by Polyacrylamide Gel Electrophoresis and Western Blotting

Caspase-3 and LC3 analysis by polyacrylamide gel electrophoresis and Western blotting were carried out as previously described [60]. Cells washed in PBS were lysed for 30 min on ice in a RIPA buffer (10 mM Tris-HCl, pH 7.2, 150 mM NaCl, 0.5% Nonidet NP40, 0.5% Na deoxycholate, 0.1% SDS, 2 mM EDTA and 50 mM NaF) containing a complete protease inhibitor cocktail (Roche Diagnostics, Indianapolis, IN, USA) diluted 1/25. Stock solution (25×) of complete protease inhibitor cocktail was prepared by diluting one tablet in 2 mL of distilled water. Cell lysates were cleared by a 20 min centrifugation at 20,000× *g*. The protein concentration was measured in the supernatant using the Bicinchoninic Acid Assay (Sigma-Aldrich). Seventy micrograms of protein were diluted in loading buffer (125 mM Tris-HCl, pH 6.8, 10% β-mercaptoethanol, 4.6% SDS, 20% glycerol, and 0.003% bromophenol blue), separated on a 14% SDS-PAGE gel for caspase-3 and LC-3, and transferred onto a nitrocellulose membrane (Bio-Rad). After blocking nonspecific binding sites for 2 h with 5% milk powder in PBST (PBS, 0.1% Tween 20, pH 7.2), the membrane was incubated overnight at 4 °C with the primary antibody diluted in PBST. The antibodies raised against caspase-3 (rabbit polyclonal antibody; Ozyme/Cell Signaling (Montigny-le-Bretonneux, France); ref: #9662; detecting endogenous levels of full length caspase-3 (35 kDa) and the large fragment of caspase-3 resulting from cleavage (17 kDa)), and LC3-I/II (rabbit polyclonal antibody; Sigma-Aldrich, ref: L8918; detecting LC3-I (18 kDa) and LC3-II (16 kDa)) were used at a final dilution of 1/1000. Antibody directed against β-actin (mouse monoclonal antibody; ref: A2228; Sigma-Aldrich) was used at a final concentration of 1/10,000. The membrane was then washed twice with PBST and incubated for 1 h at room temperature (around 21 °C) with horseradish peroxidase-conjugated goat anti-rabbit (Cell Signaling, #7074, Danvers, MA, USA) diluted at 1/5000. The membrane was washed with PBST and revealed using an enhanced chemiluminescence detection kit (Supersignal West Femto Maximum Sensitivity Substrate, Thermo-Scientific) and Chemidoc XRS^+^ (Bio-Rad). The level of cleaved caspase-3 was determined versus actin, and the ratio LC3-II/LC3-I was calculated with Image Lab software (Bio-Rad).

### 4.15. Statistical Analyses

The experimental data represent the mean ± standard deviation. Statistical analyses were performed using SPSS 18 software. The Mann–Whitney *U* test was used to compare the different groups, and data were considered statistically different at a *p* value of 0.05 or less.

## 5. Conclusions

Argan oil contains numerous molecules (fatty acids, polyphenols, phytosterols, and tocopherols) which have the ability to cross the blood-brain barrier and which have been shown to attenuate numerous side effects associated with neurodegeneration: oxidative stress, organelle dysfunctions and cell death. In addition, the different compounds present in argan oil could act synergistically to counteract the toxic effects of 7KC, which is formed by cholesterol auto-oxidation and identified at increased levels in the plasma, the cerebrospinal fluid and the brain of patients with some neurodegenerative diseases, especially Alzheimer’s disease [36,139]. Altogether, these data support the notion that a diet that includes argan oil or functional foods enriched with argan oil or with some of its specific compounds, which can be synthetized [140], may have beneficial effects in preventing or slowing the development of some neurodegenerative diseases.

## Figures and Tables

**Table 1:** Fatty acids content of dietary argan oils (Agadir, Berkane; Morocco) and extra virgin olive oil (Madhia, Tunisia).

Fatty Acids (mg/g of Total Lipids)	Dietary Argan Oils		Extra Virgin Olive Oil
	Morocco (Agadir)	Morocco (Berkane)	Tunisia (Mahdia)
C12:0	0.00	0.00	0.00
C14:0	1.47 ± 0.00	1.47 ± 0.00	0.00
C15:0	0.52 ± 0.01	0.59 ± 0.02	0.00
C16:0	119.00 ± 0.37	132.00 ± 4.97	198.00 ± 6.51
C16:1 n-7	1.04 ± 0.03	1.06 ± 0.05	29.00 ± 1.00
C16:1 n-9	0.00	0.00	1.00 ± 0.00
C17:0	0.84 ± 0.04	0.97 ± 0.02	0.00
C18:0	62.60 ± 1.27	65.00 ± 2.70	24.70 ± 1.53
C18:1 *trans*	10.90 ± 2.70	10.70 ± 0.40	10.00 ± 2.00
C18:1n-9 (oleic acid)	443.00 ± 0.51	464.00 ± 18.40	471.00 ± 15.00
C18:1 n-7	3.86 ± 0.075	4.01 ± 0.01	38.00 ± 100.00
C18:2 n-6 *cis trans*	0.00	0.00	0.00
C18:2 n-6 *trans cis*	0.00	0.00	0.00
C18:2 n-6 (linoleic acid)	332.00 ± 5.72	324.00 ± 9.21	190 ± 3.51
C20:0	3.23 ± 0.10	3.66 ± 0.09	4.00 ± 0.00
C20:1 n-9	2.95 ± 0.01	3.38 ± 0.06	2.00 ± 0.00
C18:3 n-3	1.16 ± 0.04	1.14 ± 0.01	7.33 ± 0.58
C20:2 n-6	0.00	0.00	0.00
C22:0	1.11 ± 0.00	1.34 ± 0.02	1.00 ± 0.00
C22:1 n-9	0.00	0.00	0.00
C24:0	0.53 ± 0.02	0.00	0.00
C24:1 n-9	0.00	0.00	0.00
Conjugated C18:3	0.00	0.00	0.00

**Table 2:** Sterols content of dietary argan oils (Agadir, Berkane; Morocco) and extra virgin olive oil (Madhia, Tunisia).

Sterol Contents (mg/kg of Oil)	Dietary Argan Oils		Extra Virgin Olive Oil
	Morocco (Agadir)	Morocco (Berkane)	Tunisia (Madhia)
Cholesterol	ND	ND	ND
Brassicasterol	ND	ND	ND
24-Methylenecholesterol	ND	ND	10.90 ±1.05
Campesterol	ND	ND	75.60 ± 7.14
Campestanol	16.40 ± 6.76	15.90 ± 7.11	11.30 ± 0.6.1
Stigmasterol	ND	ND	15.20 ± 0.31
∆7-Stigmasterol	48.20 ± 18.70	46.60 ± 9.31	ND
∆7-Campesterol	37.50 ± 12.70	35.30 ±28.00	ND
Spinasterol	64.40 ± 249.00	567.00 ±74.60	ND
Clerosterol	ND	ND	27.40 ± 0.61
β-Sitosterol	ND	ND	1700.00 ± 32.50
∆5-Avenasterol	ND	ND	203.00 ± 2.75
β-amyrine	211.00 ± 130.00	178.00 ± 13.00	20.10 ± 0.40
Fucosterol	ND	ND	20.20 ± 1.40
Graminasterol	ND	ND	29.00 ± 20.00
Schottenol	849.00 ± 402.00	62.50 ± 96.40	ND
Cycloartenol	239.00 ± 51.40	218.00 ± 46.80	272.00 ± 7.69
∆7-avenasterol	85.80 ± 47.10	57.8 ± 3.00	12.30 ± 1.35
24-Methylene cycloartenol	51.50 ± 27.00	44.50 ±22.30	333.30 ± 69.00
Lupeol	15.60 ± 7.13	22.70 ± 5.77	ND
Citrostadienol	132.00 ± 61.60	70.30 ± 14.00	202.00 ± 7.44
Unkown	133.00 ± 42.50	135.00 ± 204.00	29.30 ± 4.45

ND: not detected.

**Table 3:** Tocopherols contents of dietary argan oils (Agadir, Berkane; Morocco) and extra virgin olive oil (Madhia, Tunisia).

Tocopherols (mg/kg of Oil)	Dietary Argan Oils		Extra Virgin Olive Oil
	Morocco (Agadir)	Morocco (Berkane)	Tunisia (Mahdia)
α-tocopherol	429.00 ± 7.00 (995.35 ± 16.24 µM)	12.00 ± 1.00 (27.84 ± 2.32 µM)	112.00 ± 3.00 (259.86 ± 6.96 µM)
γ-tocopherol	12.00 ± 1.00 (28.77 ± 2.39 µM)	355.00 ± 19.00 (851.31 ± 45.56 µM)	9.00± 2.00 (21.58 ± 4.79 µM)
δ-tocopherol	12.00 ± 1.00 (29.77 ± 2.48 µM)	18.00 ± 2.00 (44.66 ± 4.96 µM)	0.00 (0.00 µM)
Ratio [(α-tocopherol)/(γ-tocopherol)]	35.75	0.03	12.44

The molecular weight used to calculate the concentrations are the following: α-tocopherol (431 g/mol, γ-tocopherol (417 g/mol), and δ-tocopherol (403 g/mol).

**Table 4:** Polyphenols content of dietary argan oils (Agadir, Berkane; Morocco) and extra virgin olive oil (Madhia, Tunisia).

Polyphenols	Dietary Argan Oils		Extra Virgin Olive Oil
	Morocco (Agadir)	Morocco (Berkane)	Tunisia (Mahdia)
Homovanillic acid	ND	ND	ND
Vanillin	ND	ND	ND
p-Coumaric acid	ND	ND	0.3
Quercetin-3β-glucoside	ND	ND	ND
Quercetin	ND	ND	ND
Apigenin	ND	ND	ND
2.6-dihydroxybenzoic acid	ND	ND	ND
Chlorogenic acid	ND	ND	ND
Ferrulic acid	ND	ND	0.11
Thymoquinone	ND	ND	ND
Hydroxytyrosol	ND	ND	0.65
Tyrosol	0.07	0.06	1.22
Oleuropein	ND	ND	0.39
Luteoline	ND	ND	0.39
Protocatechic acid	ND	0.17	ND
Sum of identified pics	0.07	0.18	2.78
Sum of 280 nm pics	0.54	0.75	4.62

Polyphenol content is expressed as mg equivalents quercetin/100 g of oil; ND: Not Detected.

**Table 5:** Kit Radicaux Libres (KRL) and ferric reducing antioxidant power (FRAP) assays for estimating antioxidant activities.

Compounds	Antioxidant Activity (Trolox Equivalent)	
	KRL	FRAP
α-tocopherol	0.96 ± 0.01 *	0.86 ± 0.03 *
Argan oil (Agadir)	6372 ± 318 ^#^	7463 ± 373 ^#^
Argan oil (Berkane)	7524 ± 376 ^#^	6372 ± 318 ^#^
Extra virgin olive oil (Madhia, Tunisia)	7480 ± 374 ^#^	7524 ± 376 ^#^

* Data are presented in Trolox Equivalent: one mole of α-tocopherol is equivalent to X mole (values shown in the Table) of Trolox. ^#^ Data are presented in Trolox Equivalent: 1 mL of oil is equivalent to X mole (values shown in the Table) of Trolox. Data shown are mean of three independent experiments realized in triplicate.

## Supplementary Materials

The following are available online at www.mdpi.com/1422-0067/18/10/2220/s1.
