# Supplementary material for: Argan Oil-Mediated Attenuation of Organelle Dysfunction, Oxidative Stress and Cell Death Induced by 7-Ketocholesterol in Murine Oligodendrocytes 158N
Source: Int J Mol Sci. 2017 Oct 23;18(10):2220. doi: 10.3390/ijms18102220 (PMC5666899; doi:10.3390/ijms18102220)
Supplement: Supplementary file 1 [file ijms-18-02220-s001.pdf]

International Journal of *Molecular Sciences*

MDPI

Article

Argan Oil-Mediated Attenuation of Organelle Dysfunction, Oxidative Stress and Cell Death Induced by 7-Ketocholesterol in Murine Oligodendrocytes 158N

Asmaa Badreddine [1,2], Amira Zarrouk [3,4], El Mostafa Karym [1,2], Meryam Debbabi [1,3], Thomas Nury [1], Wiem Meddeb [1,5], Randa Sghaier [1,4], Maryem Bezine [1,6], Anne Vejux [1], Lucy Martine [7], Stéphane Grégoire [7], Lionel Bretillon [7], Emmanuelle Prost-Camus [8], Philippe Durand [8], Michel Prost [8], Thibault Moreau [9], Mustapha Cherkaoui-Malki [1], Boubker Nasser [2] and Gérard Lizard [1,*]

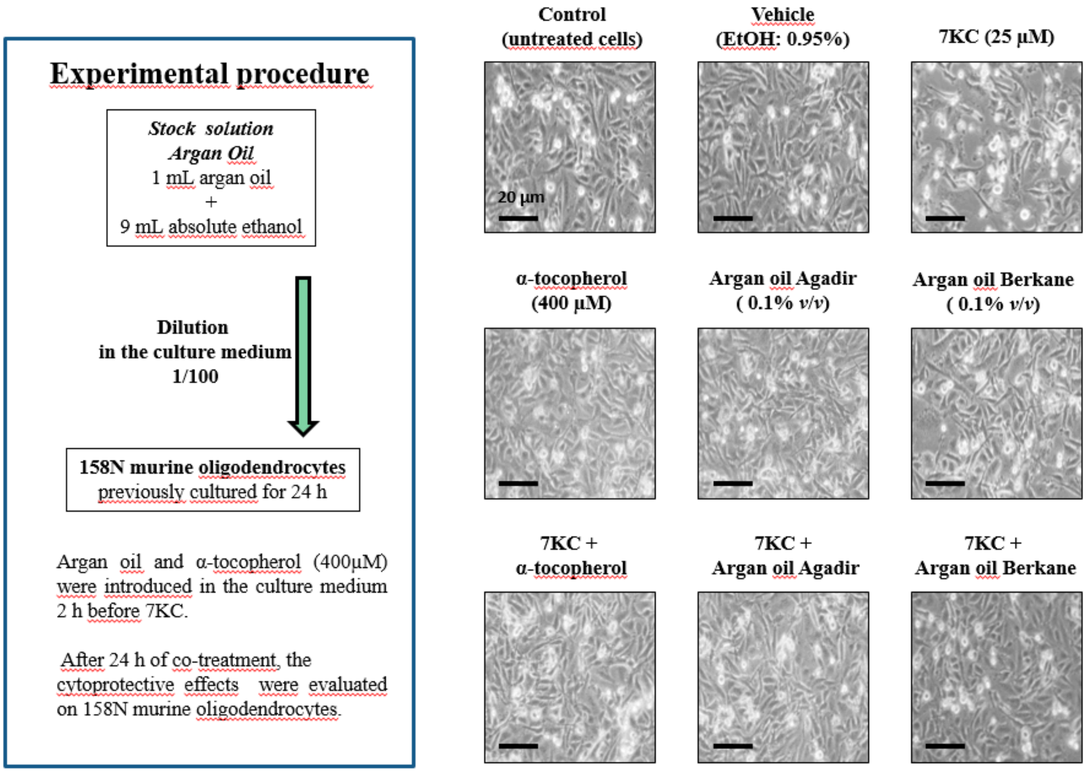

Figure S1. Procedure of evaluation of the biological activity of argan oil on 7-ketocholesterol-treated 158N murine oligodendrocytes: data obtained by phase contrast microscopy. After 24 h of culture, 158N murine oligodendrocytes were cultured for an additional 24 h without or with 7KC (25 µM) in the absence or presence of argan oils (Berkane or Agadir; Morocco; 0.1% *v*/*v*) or of α-tocopherol (400 µM) used as the positive control. Argan oil from Agadir and Berkane was prepared and added on 158N cells as summarized in the left part of the figure. The cytoprotective effect of argan oils on 7KC-induced inhibition of cell growth was evaluated by phase-contrast microscopy. A: control (untreated cells); B: vehicle (EtOH) 0.9% *v*/*v*; C: 7KC (25 µM)-treated cells; D: dietary argan oil (Agadir 0.1% *v*/*v*); E: dietary argan oil (Berkane 0.1% *v*/*v*); F: α-tocopherol: 400 µM; G: 7KC (25 µM) + dietary argan oil (Agadir 0.1% *v*/*v*); H: 7KC (25 µM) + dietary argan oil (Berkane 0.1% *v*/*v*); J: 7KC (25 µM) + α-tocopherol (400 µM). The pictures shown are representative of at least three independent experiments. The scale bars are 20 µm.
